# Raman spectroscopy as probe of nanometre-scale strain variations in graphene

**DOI:** 10.1038/ncomms9429

**Published:** 2015-09-29

**Authors:** C. Neumann, S. Reichardt, P. Venezuela, M. Drögeler, L. Banszerus, M. Schmitz, K. Watanabe, T. Taniguchi, F. Mauri, B. Beschoten, S. V. Rotkin, C. Stampfer

**Affiliations:** 1 JARA-FIT and 2nd Institute of Physics, RWTH Aachen University, Aachen 52074, Germany; 2 Peter Grünberg Institute (PGI-9), Forschungszentrum Jülich, Jülich 52425, Germany; 3 Instituto de Fsica, Universidade Federal Fluminense, Niterói, 24210-346 Rio de Janeiro, Brazil; 4 National Institute for Materials Science,1-1 Namiki, Tsukuba 305-0044, Japan; 5 IMPMC, UMR CNRS 7590, Sorbonne Universités—UPMC Univ. Paris 06, MNHN, IRD, 4 Place Jussieu, Paris 75005, France; 6 Department of Physics and Center for Advanced Materials and Nanotechnology, Lehigh University, Bethlehem, Pennsylvania 18015, USA

## Abstract

Confocal Raman spectroscopy has emerged as a major, versatile workhorse for the non-invasive characterization of graphene. Although it is successfully used to determine the number of layers, the quality of edges, and the effects of strain, doping and disorder, the nature of the experimentally observed broadening of the most prominent Raman 2D line has remained unclear. Here we show that the observed 2D line width contains valuable information on strain variations in graphene on length scales far below the laser spot size, that is, on the nanometre-scale. This finding is highly relevant as it has been shown recently that such nanometre-scaled strain variations limit the carrier mobility in high-quality graphene devices. Consequently, the 2D line width is a good and easily accessible quantity for classifying the crystalline quality, nanometre-scale flatness as well as local electronic properties of graphene, all important for future scientific and industrial applications.

## Abstract (web summary)

Raman spectroscopy has become an invaluable tool for graphene characterisation, yet the nature of the broadening of the Raman 2D line remains unclear. Here, Stampfer *et al*. show that the Raman 2D line width is a measure of nanometre-scale strain variations in graphene on insulating substrates.

## Graphical abstract

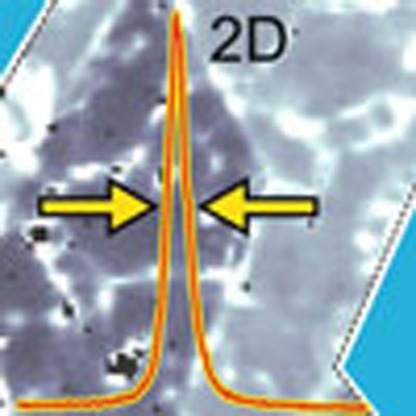

Graphene combines several highly interesting material properties in a unique way, promising unprecedented material functionality. This makes graphene increasingly attractive for fundamental research as well as industrial applications^1^, but, at the same time, stresses the need for non-invasive characterization techniques. In recent years, Raman spectroscopy has proven to be highly useful as a non-invasive method not only to identify graphene^2^,^3^ but also to extract information on doping^4^,^5^,^6^,^7^, strain^8^,^9^ and lattice temperature^10^,^11^. Even more insights can be gained when utilizing confocal, scanning Raman spectroscopy to study spatially resolved doping domains^7^,^12^, edge effects^3^,^13^ and position-dependent mechanical lattice deformations, including strain^14^,^15^,^16^. The spatial resolution of so-called Raman maps is on the order of the laser spot size (which for confocal systems is typically on the order of 500 nm) and the extracted quantities (such as doping or strain) are in general averaged over the spot size. It is therefore important to distinguish between length scales significantly larger or smaller than the laser spot size. In particular, we will distinguish between strain variations on a micrometre scale, which can be extracted from spatially resolved Raman maps, and nanometre-scale strain variations, which are on sub-spot-size length scales and cannot be directly observed. Especially, nanometre-scale strain variations have been recently identified as the most important limitation to carrier mobility in high-quality graphene^17^, making this quantity increasingly important^18^.

In this article, we show that the experimentally observed Raman 2D line width is a measure of nanometre-scale strain variations in graphene on insulating substrates, that is, it contains valuable information on local (that is, nanometre-scale) flatness, lattice deformations and crystal quality of graphene. Our findings solve the long-standing question of the nature of the observed broadening of the Raman 2D line and also link this quantity to the electronic transport properties of graphene, making it a valuable quantity for classifying the quality of graphene. To prove that the experimentally observed 2D line width depends on sub-spot-size strain variations and lattice deformations, we employ the following strategy.

We start by showing that by combining Raman spectroscopy with magnetic fields, electronic broadening contributions for the Raman G line width can be strongly suppressed. Since in perpendicular magnetic fields the electronic states in graphene condense into Landau levels (LLs), the interaction between electronic excitations and lattice vibrations becomes *B*-field dependent. In agreement with existing theory^19^,^20^,^21^,^22^ and experiments^23^,^24^, we demonstrate that by applying a perpendicular *B*-field of ∼8 T, the G line becomes almost independent of electronic properties such as charge carrier doping, screening, or electronic broadening.

We observe that, under these conditions, the G line width nevertheless exhibits strong variations across graphene flakes. In particular, we show that the G line width is significantly increased in regions where the graphene flake features bubbles and folds, that is, in correspondence with increased structural deformations.

Finally, we show that at 8 T, there is a (nearly) linear dependence between the G line width and the 2D line width, implying that there is a common source of line broadening. According to the previous points, the broadening must be related to structural lattice deformations. This finding is further supported by a detailed analysis of the relation between the area of the 2D peak and its line width. By analysing the relation between the G and 2D line width, we find that nanometre-scale strain variations constitute a dominant contribution to the observed line broadenings. Importantly, the 2D line has been shown to only very weakly depend on the *B*-field^25^, implying that no magnetic field is required to extract information on nanometre-scale strain variations from the 2D line width, which makes this quantity interesting for practical applications.

## Results

### Sample characterization

**Figure 1:**
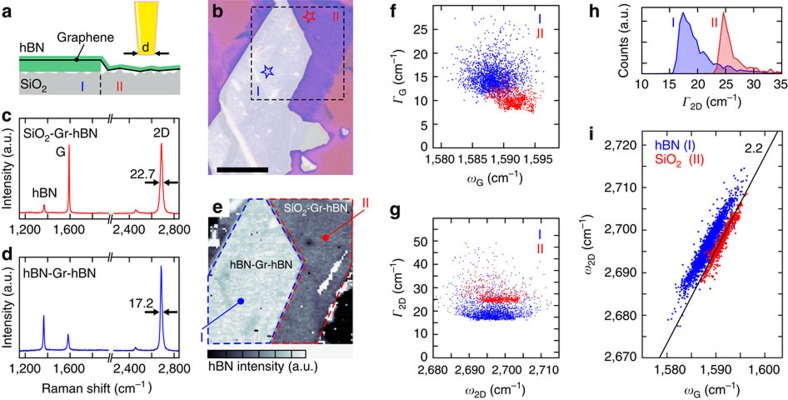
Graphene sample characterization. (**a**) Schematic representation of cross-section of the investigated sample highlighting the different regions I (hBN-Gr-hBN) and II (SiO_2_-Gr-hBN). (**b**) Optical image of a Gr-hBN heterostructure resting partly on hBN and SiO_2_. Scale bar, 10 μm. (**c**,**d**) Raman spectrum taken on the SiO_2_-Gr-hBN (**c**) and hBN-Gr-hBN (**d**) areas. The positions where the spectra were taken are marked by a blue and a red star, respectively (**b**). (**e**) Raman map of the intensity of the hBN peak. The dashed lines mark the regions I and II. (**f**) Γ_G_ versus *ω*_G_ recorded on various spots on regions I (blue) and II (red) of the sample. (**g**) Γ_2D_ versus *ω*_2D_ recorded on various spots on regions I (blue) and II (red) of the sample. (**h**) Histograms of Γ_2D_ recorded on various spots on regions I (blue) and II (red) of the sample. (**i**) *ω*_2D_ versus *ω*_G_ recorded on various spots on regions I (blue) and II (red) of the sample.

The investigated graphene (Gr) sheet is partly encapsulated in hexagonal boron nitride (hBN) and partly sandwiched between SiO_2_ and hBN as illustrated in Fig. 1a. An optical image of our sample is shown in Fig. 1b. In contrast to graphene encapsulated in hBN, graphene flakes supported by SiO_2_ usually feature lower carrier mobilities of around 10^3^–10^4^ cm^2^/(Vs), indicating a detrimental influence of SiO_2_ on the electronic properties of graphene. In this regard, our structure gives us the invaluable capability of probing a single graphene sheet exposed to two different substrates (regions I and II in Fig. 1a,b). The sample is fabricated with a dry and resist-free transfer process following Wang *et al*.^26^ and Engels *et al*.^27^, where we pick up an exfoliated graphene flake with an hBN flake and deposit it onto the hBN-SiO_2_ transition area of the substrate. A typical Raman spectrum of graphene supported by SiO_2_ and covered by hBN, taken at the position of the red star in Fig. 1b, is shown in Fig. 1c. The characteristic hBN line as well as the graphene G and 2D lines can be clearly identified. At first glance, the spectra recorded in the hBN-Gr-hBN area look similar (see Fig. 1d, taken at the position marked by the blue star in Fig. 1b). However, it is evident that the full-width at half-maximum (FWHM) of the 2D line, Γ_2D_, is significantly smaller.

The confocal nature of our Raman setup enables us to do spatially resolved measurements. An example of a Raman map is shown in Fig. 1e, where the spatially resolved intensity of the hBN line is depicted. The hBN and SiO_2_ areas can be clearly distinguished in the map (see highlighted regions I and II). While analysing the Raman spectra of every point on the map, it is evident that the G lines recorded in the hBN-encapsulated area are broader than in the SiO_2_ supported area (compare red and blue data points in Fig. 1f). This is a clear indication of reduced charge carrier doping induced by the hBN substrate compared with SiO_2_. In fact, at low charge carrier doping, the phonon mode can decay into electron–hole pairs, which results in a broadening of the G peak^5^,^28^. For the 2D line, in contrast, the Γ_2D_ recorded in the hBN-encapsulated area is mostly between 17 cm^−1^ and 20 cm^−1^, while it is above 22 cm^−1^ in the SiO_2_ area (see blue and red curves in the histogram of Fig. 1h, respectively). Note that both Γ_2D_ and Γ_G_ do not depend on the respective frequencies *ω*_2D_ and *ω*_G_ (Fig. 1f,g). In Fig. 1i the position of the G and 2D lines for every spectrum obtained on the investigated graphene sheet are displayed. For both substrates, the data points scatter along a line with a slope of 2.2. This slope coincides with the ratio of strain-induced shifts (that is, of the related Grüneisen parameters) of the Raman G and 2D modes^29^. This indicates that there are significant strain variations on both substrates across the entire graphene layer. Assuming the strain to be of biaxial nature, the spread of the data points translates into a maximum, micrometre-scale strain variation of ∼0.14% (ref. 29). The offset of the SiO_2_ and hBN data points can be understood in terms of the higher charge carrier doping induced by the SiO_2_ substrate, which shifts the data points towards higher values of *ω*_G_ (ref. 5), and differences in the dielectric screening of hBN and SiO_2_ that effectively shift the 2D line position^30^. Since the data stems from a single graphene flake that has undergone identical fabrication steps for both substrate regions, the difference in charge carrier doping is unambiguously because of the two different substrate materials.

### Suppressing electronic broadening with a magnetic field

**Figure 2:**
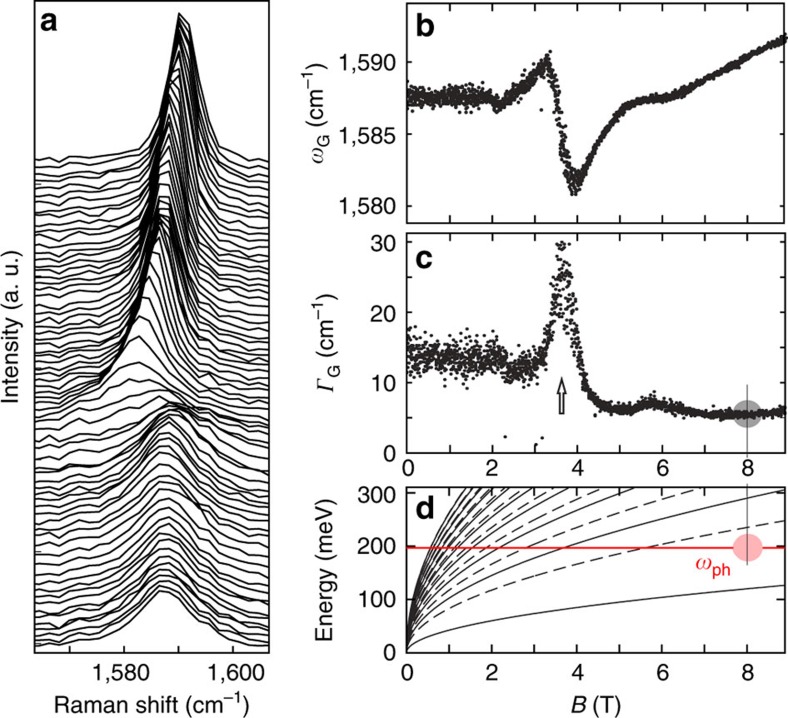
Magneto-Raman spectroscopy. (**a**) Raman spectra recorded as a function of magnetic field, ranging from 0 T (bottom spectrum) to 8.9 T (top spectrum). The spectra are vertically offset for clarity. (**b**,**c**) Frequency, *ω*_G_, and FWHM, Γ_G_, of the G peak as a function of magnetic field as obtained from Lorentzian fits to the data shown in **a**. The arrow (**c**) highlights a value of the magnetic field at which the phonon is energetically matched to a LL transition. (**d**) Evolution of the energies of LL transitions with magnetic field. The full lines represent inter-band transitions in which the LL index changes by one. The dashed lines represent inter-band transitions in which the LL index does not change. The red line represents the G mode phonon frequency at zero *B* field. The circled region in (**c**,**d**) highlights the region in which no LL transitions energetically match the G mode phonon.

For a more refined comparison of the Raman spectra on both substrates, we seek to suppress the effects on the G line arising from these differences in charge carrier doping. We therefore minimize the influence of the electronic system on the Raman G line by applying a perpendicular magnetic field. In the presence of a perpendicular magnetic field, the electronic states in graphene condense into LLs. The coupling of these LLs to the G mode is well understood^19^,^20^ and experimentally confirmed^22^,^23^,^24^,^31^,^32^,^33^,^34^,^35^,^36^. When a LL transition energetically matches the G mode phonon, the position of the G line is shifted and its line width increases. An example for the evolution of the Raman G peak with magnetic field, taken on the hBN sandwich area, is shown in Fig. 2a. The individual spectra are offset for clarity. For a detailed analysis, single Lorentzians are fitted to every spectrum. The resulting frequency, *ω*_G_, and FWHM, Γ_G_, are displayed in Fig. 2b,c, respectively. The arrow at *B*=3.7 T (Fig. 2c) shows a value of the magnetic field where a LL transition is energetically matched with the phonon, leading to a broadening of the G line. However, at magnetic fields around 8 T, no LL transition is energetically close to the G mode, as illustrated in Fig. 2d, where the energies of the relevant LL transitions as a function of magnetic field are compared with the energy of the G mode phonon. Consequently, at this high magnetic field the influence of the electronic system on the position and width of the G line is minimized. Note that this effect is independent of the charge carrier density and the exact values of the broadening of the LL transitions assuming that the latter are within a reasonable range as found by other studies^24^,^35^. Thus, the residual broadening of the G line is most likely determined by phonon–phonon scattering and averaging effects over different strain values that vary on a nanometre scale.

### Strain variations within the laser spot

**Figure 3:**
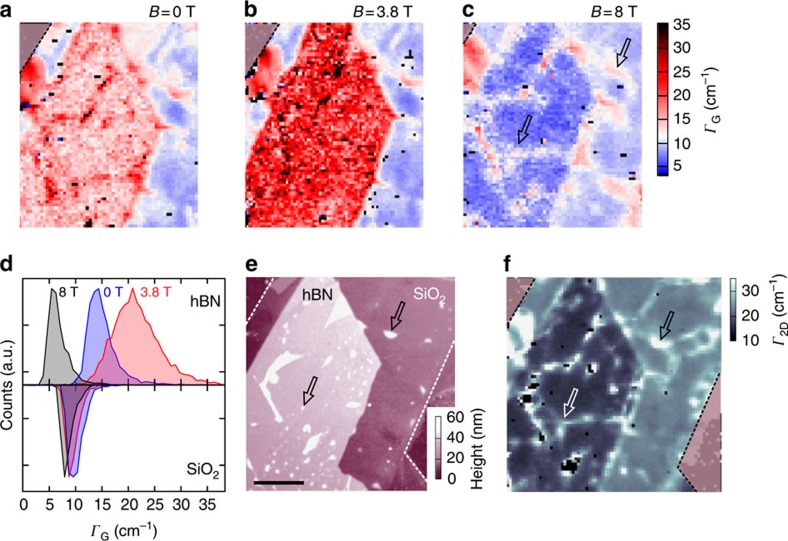
Sample morphology probed by Raman spectroscopy. (**a**–**c**) Raman maps of the FWHM of the G peak, Γ_G_, taken at different magnetic fields, *B*=0 T (**a**), 3.8 T (**b**) and 8 T (**c**). The different regions I and II (labelled in Fig. 1a) can be well distinguished in all three panels. (**d**) Histograms of Γ_G_ for the different magnetic fields, *B*=0 T (blue), 3.8 T (red), *B*=8 T (grey) and the two substrates hBN (top panel) and SiO_2_ (bottom panel). (**e**) SFM image of the investigated sample. The scale bar represents 5 μm. (**f**) Raman map of Γ_2D_ recorded at 0 T. The arrows highlight mechanical folds visible in the SFM image as well as in the Raman maps (**c**,**e**,**f**). SFM, scanning force microscopy.

To demonstrate that this applies to the entire sample, we first show that the broadening of the electronic states is low enough on the entire hBN-Gr-hBN area. In Fig. 3a,b, we show maps of Γ_G_ at *B*=0 and 3.8 T, respectively. On the hBN part, the width of the G line shows the resonant behaviour depicted in Fig. 2c (see also histogram in Fig. 3d). This effect happens throughout the entire hBN area, independent of the local doping and strain values and independent of possible local folds and bubbles. The suppression of magneto-phonon resonances on the SiO_2_ substrate can be attributed to the higher charge carrier density. At higher charge carrier density the needed LL transitions are blocked by the Pauli principle. In the next step, we tune the magnetic field to 8 T, where the electronic influences on the Raman G line are at a minimum. A map of Γ_G_ over the entire flake at a magnetic field of 8 T is shown in Fig. 3c. Distinct features across the whole sample are visible as regions with increased line width. A comparison with a scanning force microscope image of the sample (Fig. 3e) reveals that many of these regions can be associated with folds and bubbles most likely induced during the fabrication process, some of which even cross the border between the underlying hBN and SiO_2_ substrate regions.

As electronic broadening effects are suppressed at 8 T, the increased line width of the G line in the vicinity of these lattice deformations arises from enhanced phonon–phonon scattering and/or an averaging effect over varying nanometre-scale strain conditions.

**Figure 4:**
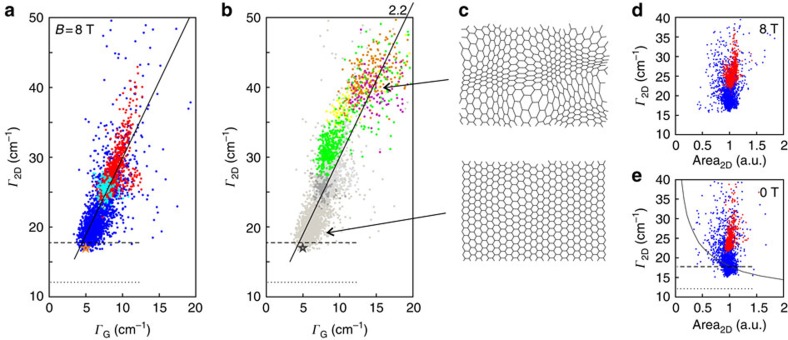
Nanometre-scale strain variations. (**a**) Γ_G_ versus Γ_2D_ recorded on various points on the hBN part (blue) and SiO_2_ (red) of the sample at a magnetic field of 8 T. Additional data points from a graphene-on-SiO_2_ sample (teal) and a second hBN-Gr-hBN sample (orange star) are shown. (**b**) The data points of (**a**) are depicted in grey. The coloured data are recorded on four different CVD graphene flakes on SiO_2_ substrate at 0 T. All four samples have doping values of *n*_el_>3 × 10^12^ cm^−2^, such that Landau damping of the G line is suppressed. The dashed and dotted lines in (**a**,**b**) indicate the calculated values of Γ_2D_ from DFT calculations including electron–phonon and phonon–phonon broadening (dotted line) and electron–phonon, electron–eletron and phonon–phonon broadening (dashed line). (**c**) Two schematic illustrations of nanometre-scale strain variations (top: large variations; bottom: small variations). (**d**) Γ_2D_ versus the integrated area of the 2D peak as obtained from single Lorentzian fits for the hBN part (blue) and SiO_2_ (red) measured at 8 T. Both data clouds are scaled to an average area_2D_ value of one. (**e**) Similar plot as in panel (**d**) but for 0 T. The solid black line is the calculated dependence of Γ_2D_ and area_2D_ for varying electronic broadening from the first-principles calculations, specified in the text and in Venezuela *et al*.^38^. The dashed and dotted black lines are the same as in (**a**,**b**). DFT, density-functional theory.

Interestingly, the same features can also be identified in a Γ_2D_ map recorded at *B*=0 T, shown in Fig. 3f. This strongly suggests that the lattice deformations identified at 8 T in Γ_G_ also cause a broadening of the 2D mode. The same trend is highlighted in Fig. 4a, where we show the relation of Γ_G_ and Γ_2D_ for all recorded Raman spectra at 8 T. The additional teal data points stem from a Gr-SiO_2_ sample and the orange star originates from a different hBN-Gr-hBN sandwich structure with all data having been obtained at 8 T. Notably, the points from all substrate regions lie on one common line. From this linear relation between Γ_2D_ and Γ_G_ (Fig. 4a), we conclude that there must be a common source of line broadening, which is connected to structural deformations. This is mainly due to the fact that at 8 T the G-line broadening is only very weakly affected by electronic contributions (see above). The range of the presented scatter plot can be extended by including data recorded on low-quality graphene samples with significant doping, as shown in Fig. 4b. Here, no magnetic field but high doping (corresponding to Fermi energies much higher than half of the phonon energy ℏ*ω*_ph_/2≈100 meV) is used to suppress Landau damping of the G mode, leaving Γ_G_ unaffected from electronic contributions. The coloured data points stem from Raman maps (*B*=0 T) of chemical vapour deposition (CVD)-grown graphene flakes that were transferred onto SiO_2_ by a wet chemistry-based transfer. These graphene sheets contain doping values of *n*_el_>3 × 10^12^ cm^−2^, which corresponds to Fermi energies *E*_F_>200 meV (Supplementary Figs 1 and 2). The data points show the same trend as the values obtained at 8 T (grey data points in Fig. 4b) and even extend the total range of the dependence to higher values of Γ_2D_.

## Discussion

**Figure 5:**
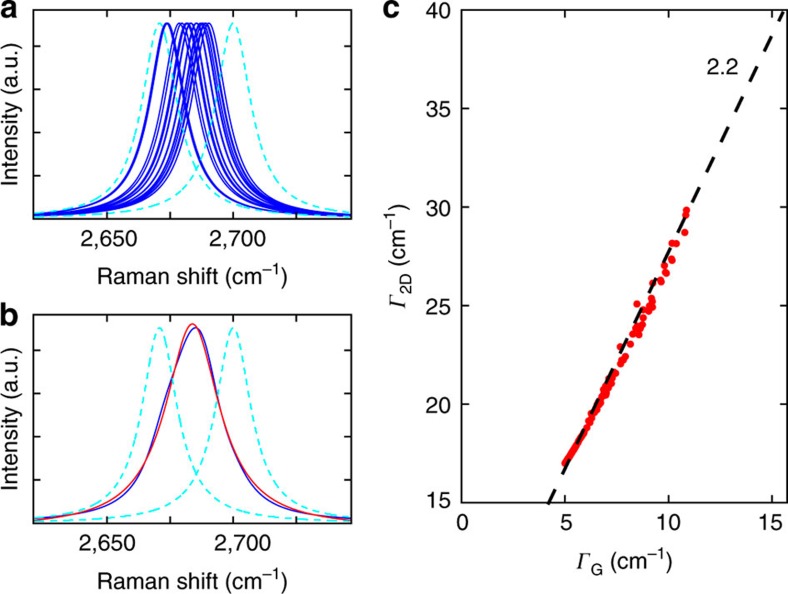
Illustration of line broadening due to averaging effects. (**a**,**b**) Individual Raman processes within the laser spot (**a**) add up to a broad Raman peak that is recorded (blue peak in **b**). The red curve in (**b**) is a Lorentzian fit to the blue line. The dashed cyan lines, representing the two outmost individual Raman processes, are the same in (**a**,**b**) and serve as a guide to the eye. (**c**) Width of the statistical broadened Raman G and 2D peaks as obtained from a simple statistical model. The procedure described in (**a**,**b**) was performed for different sets of strain variations. Each set of strain values follows a Gaussian distribution centred at 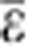
=−0.1% with a width varying from Δ*ɛ*=0–0.15%. The resulting G and 2D peaks are fitted with a single Lorentzian with the respective widths represented by a red point. The dashed black line has the slope of 2.2. It matches to the generated data points indicating that strain variations within the laser spot broaden the G and 2D lines by this ratio.

Although the linear relation between Γ_G_ and Γ_2D_ in Fig. 4a,b shows that structural deformations also broaden the 2D line, it is less straightforward to identify the actual mechanism of broadening. In principle, it is possible that the high values of Γ_2D_ around folds and bubbles are due to a combination of increased phonon–phonon scattering, averaging effects over different strain values within the laser spot and reduced electronic life times. However, interestingly the slopes in Fig. 4a,b are around 2.2 (see black lines). This is a remarkable resemblance to the strain-induced frequency shifts of both modes (compare Fig. 1i). This provides very strong indication that averaging over different strain values, which vary on a nanometre scale (see Fig. 4c), play an important role in the broadening of the experimentally observed 2D line. This averaging effect broadens the G and 2D line by the same ratio as their peak positions shift for fixed average strain values explaining the slope of 2.2 between Γ_G_ and Γ_2D_ (Supplementary Fig. 3), which we demonstrate with a simple toy model as shown in Fig. 5. Each individual Raman process that takes place within the laser spot is subject to a different amount of strain since the latter varies across the laser spot. Each of the corresponding Raman peaks is thus shifted by a different amount (see blue and dashed cyan curves in Fig. 5a). The intrinsic broadening of each individual Raman process is assumed to be Γ_G_=5 cm^−1^ and Γ_2D_=17 cm^−1^. Due to the size of the laser spot, the sum of several of these individual Raman processes is recorded, with the resulting peak being given by the sum of the individual peaks (see blue curve in Fig. 5b). Following the data analysis of our measurements, the resulting curve is fitted by a single Lorentzian (red curve in Fig. 5b). To simulate the effect of this statistical broadening mechanism on the width of the resulting Raman peak, we simulate statistical strain distributions for several laser spots that are subject to different amounts of strain variation Δ*ɛ* in Fig. 5c. For each of the red points, 20 strain values were randomly generated. Each set of strain values follows a Gaussian distribution centred at 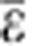
=−0.1% with a width varying from Δ*ɛ*=0 to 0.15%. The dashed black line has a slope of 2.2 and matches the distribution of the red points, illustrating that averaging over nanometre-scale variations leads to a linear dependence of Γ_G_ and Γ_2D_.

We are aware that the low charge carrier densities in the hBN-encapsulated area might result in a narrowing of the 2D mode by three to four wave numbers^37^. However, the large differences of Γ_2D_ on the order of 20–30 cm^−1^ on both substrates cannot be explained by the differences in charge carrier doping^7^,^37^,^38^.

Interestingly, the lowest Γ_2D_ observed in our experiments are very close to the value that we compute from first-principles as by Venezuela *et al*.^38^ assuming an undoped, defect-free and stress-free sample of graphene (horizontal dashed and dotted lines in Fig. 4a,b). In such an approach, the width of the 2D peak is determined by the anharmonic decay rate of the two phonons involved (5.3 cm^−1^ according to Paulatto *et al*.^39^), and, indirectly, by the broadening of the electron and hole, denoted as γ in Venezuela *et al*.^38^ (see also Basko^40^). According to Reference Venezuela *et al*.^38^, the electron–phonon contribution to γ is 81.9 meV for electronic states in resonance with the 2.33 eV laser-light. With such a value of γ we obtain a Γ_2D_ of 12.1 cm^−1^ (dotted lines in Fig. 4a,b,e). If, following Herziger *et al*.^41^, we double the value of γ to account for the electron–electron scattering, we obtain a Γ_2D_ of 17.9 cm^−1^ (dashed lines in Fig. 4a,b,e), in close agreement with the lowest measured values. In principle, the observed increase of Γ_2D_ with respect to its minimum value could be attributed to an increase of the electronic broadening γ, due to doping (increasing the electron–electron scattering) or to the presence of defects (increasing the electron-defect scattering)^38^,^40^,^42^. By investigating the relation between Γ_2D_ and the integrated area of the 2D peak (area_2D_) we can exclude such a hypothesis. In Fig. 4d,e we show scatter plots of Γ_2D_ versus the region-normalized area_2D_ for both *B*=8 T and *B*=0 T, highlighting the very weak *B*-field dependence of Γ_2D_. More importantly, we observe that the area of the 2D peak does not depend on Γ_2D_, contrary to what is expected in presence of a variation of the electronic broadening γ (refs 38, 40, 42). In particular the measured data does not follow the calculated dependence of Γ_2D_ on area_2D_, reported in Fig. 4e, obtained in the calculation by varying electronic broadening γ. This dismisses differences in the electronic broadening as a main mechanism for the observed variations of Γ_2D_.

Finally, our finding that the 2D line depends on nanometre-scale strain inhomogeneities is also in good agreement with high-resolution scanning tunnelling microscopy measurements, which reveal that graphene on SiO_2_ forms short-ranged corrugations, while graphene on hBN features significantly flatter areas^43^.

In summary, we showed that by using a magnetic field of 8 T to strongly suppress the influence of the electronic contributions on the Raman G line width, the latter can be used as a measure for the amount of nanometre-scale strain variations. Most importantly, we observed a nearly linear dependence between the G and 2D line widths at 8 T independent of the substrate material, indicating that the dominating source of the spread of the broadening of both peaks is the same. From the slope ΔΓ_2D_/ΔΓ_G_ of around 2.2, we deduce that averaging effects over nanometre-scale strain variations make a major contribution to this trend. Since the 2D line width shows only a very weak dependence on the *B* field, this quantity can even be used without a magnetic field to gain information on the local strain homogeneity and thus on the structural quality of graphene. These insights can be potentially very valuable for monitoring graphene fabrication and growth processes in research and industrial applications, where a fast and non-invasive control of graphene lattice deformations is of great interest.

## Methods

### Raman spectroscopy measurements

The room temperature Raman spectra were acquired using a commercial Witec system with a laser excitation of 532 nm (2.33 eV) delivered through a single-mode optical fibre, where the spot size is limited by diffraction. Using a long working distance focusing lens with a numerical aperture of 0.80, we obtained a spot size of ∼400–500 nm. For the low-temperature Raman measurements, we employ a commercially available confocal Raman setup that allows us to perform spatially resolved experiments at a temperature of 4.2 K and magnetic fields of up to 9 T. We use an excitation laser wavelength of 532 nm with a spot diameter on the sample of ∼500 nm. For detection, we use a single-mode optical fibre and a charge-coupled spectrometer with a grating of 1,200 lines mm^−1^. All measurements are performed with linear laser polarization and a × 100 objective.

### First-principles calculations

For the computation of the double-resonant Raman cross-section we employ an approach based on Fermi's golden rule generalized to the fourth perturbative order as described in detail in Reference Venezuela *et al*.^38^. In this approach, electron–light, and electron–phonon scattering matrix elements are explicitly calculated and the phonon and electronic dispersions reproduce calculations based on density-functional theory corrected with GW. Converged results are obtained using 480 × 480 and 240 × 240 grids in the Brillouin zone for the electron and phonon wave-vectors, respectively. The finite phonon life time is taken into account by broadening the Raman intensity with 5.3 cm^−1^ wide Lorentzians^39^. We varied the electron broadening (γ in eq. (5) from Venezuela *et al*.^38^) from 16.4 to 344 meV and then we determined Γ_2D_ as a function of area_2D_. For a laser energy of 2.33 eV, the electron–phonon contribution for the electronic broadening is 81.9 meV, which leads to Γ_2D_=12.1 cm^−1^. However, when we choose γ to be twice this value, to account for additional electron–electron interaction^41^, we obtain Γ_2D_ equal to 17.9 cm^−1^. These values can be understood as a theoretical expectation of the 2D line width for a perfect graphene lattice disregarding any broadening from averaging effects over different strain values within the laser spot.

## Additional information

**How to cite this article:** Neumann, C. *et al*. Raman spectroscopy as probe of nanometre-scale strain variations in graphene. *Nat. Commun.* 6:8429 doi: 10.1038/ncomms9429 (2015).

## Supplementary Material

Supplementary Information Supplementary Figures 1-3
