# Supplementary material for: Raman spectroscopy as probe of nanometre-scale strain variations in graphene
Source: Nat Commun. 2015 Sep 29;6:8429. doi: 10.1038/ncomms9429 (PMC4598719; doi:10.1038/ncomms9429)
Supplement: Supplementary Information — Supplementary Figures 1-3 [file ncomms9429-s1.pdf]

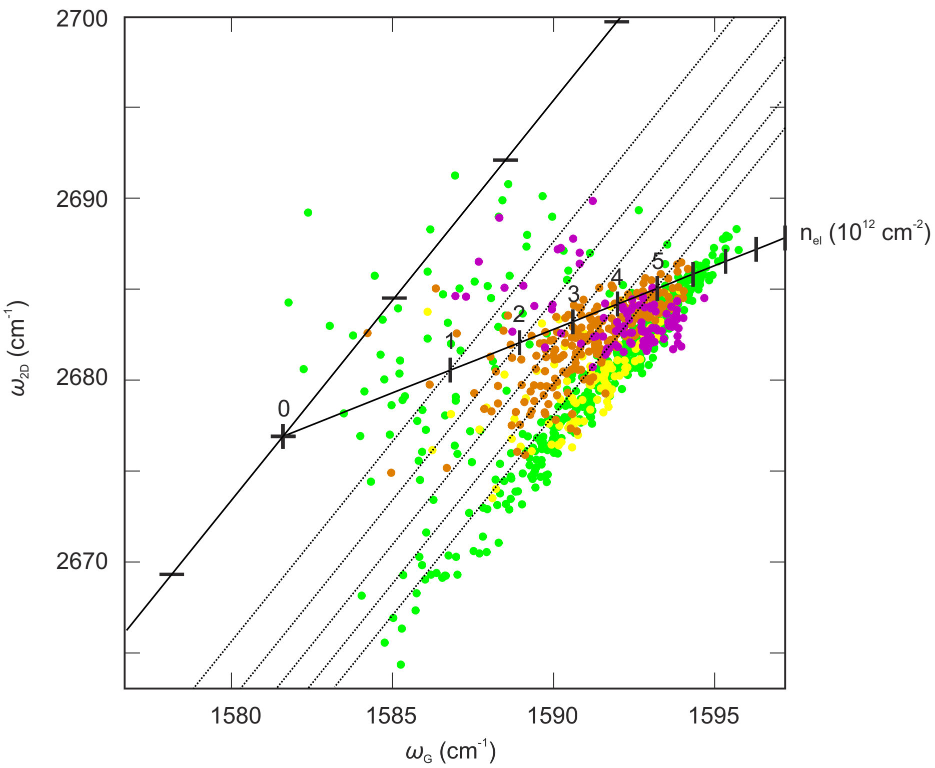

Supplementary Figure 1: Estimation of charge carrier doping of CVD-grown, wet-transferred graphene-SiO_2 flakes. G peak position, ω_G, versus 2D peak position, ω_{2D}, extracted from Raman scans of the four CVD grown samples featured in Figure 4b in the main text (colored points). The same colors are used as in Figure 4b. The thick black lines represent the axes of strain and doping induced position shifts [29]. By projecting each point onto the axis of doping induced shifts (thin black lines), one can extract the amount of average charge carrier doping within the laser spot. For all four samples shown, we find average doping values in excess of 3×10^{12} cm^{-2}, which prevents Landau damping [5] and thus switches off the effect of the electronic system on the line width of the G mode.

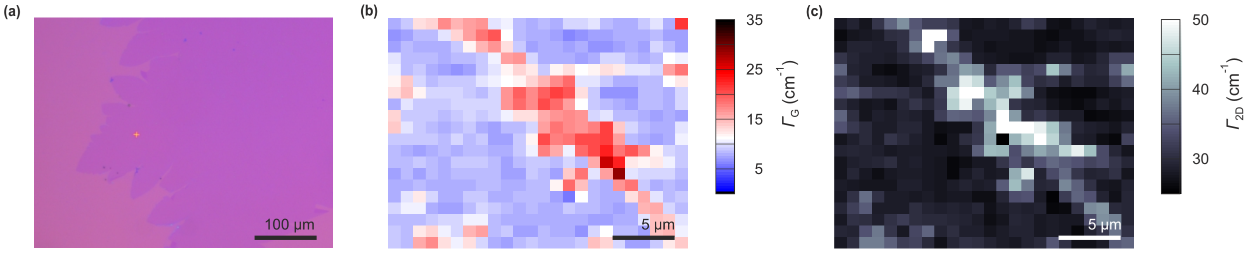

Supplementary Figure 2: Characterization of CVD-grown, wet-transferred graphene-SiO_2 samples. (a) Optical image of one of a CVD-grown, wet-transferred graphene-SiO_2 sample. (b) Raman map of Γ_G from the same sample. (c) Raman map of Γ_{2D} from the same sample. The same qualitative features are visible in both the Γ_G and Γ_{2D} maps.

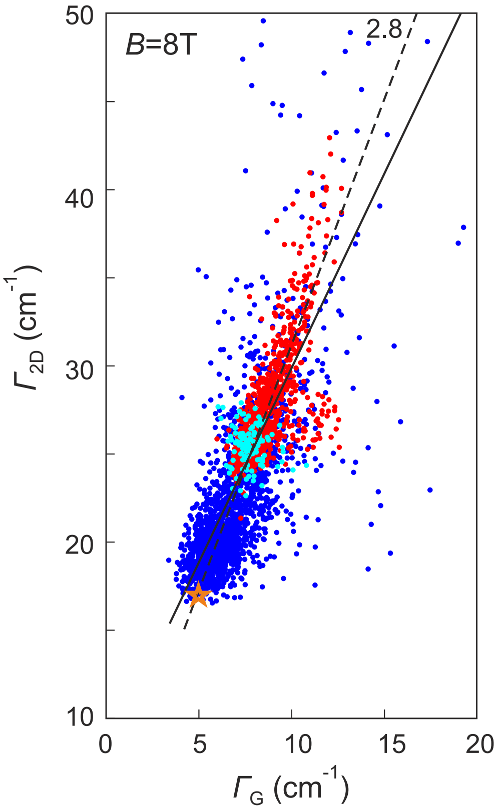

Supplementary Figure 3: Analysis of the Γ_{2D} versus Γ_G data. Linear regression (dashed line) to the data of Γ_{2D} versus Γ_G displayed in Figure 4a of the main manuscript. As the density of data points is significantly larger for low values of Γ_{2D}, we divide the Γ_{2D} axis into 40 sections in the interval of 16 cm^{-1} $< \Gamma_{2D} <$ 30 cm^{-1}. Consequently, we average Γ_{2D} and Γ_G in each section and perform a linear regression to the averaged data points. The resultant slope of 2.8 is indicated by the dashed line. The solid line is the guide to the eye with a slope of 2.2 from the main manuscript.
